# A Mitogen-Activated Protein Kinase Pathway Is Required for *Bacillus amyloliquefaciens* PMB05 to Enhance Disease Resistance to Bacterial Soft Rot in *Arabidopsis thaliana*

**DOI:** 10.3390/plants13182591

**Published:** 2024-09-16

**Authors:** Ai-Ting Li, Shang-Kai Liu, Jia-Rong Li, Sabrina Diana Blanco, Hsin-Wei Tsai, Jia-Xin Xie, Yun-Chen Tsai, Yuh Tzean, Yi-Hsien Lin

**Affiliations:** 1 Department of Plant Medicine, National Pingtung University of Science and Technology, Pingtung 912301, Taiwan; vivian12451245@gmail.com (A.-T.L.); skliou.tarm@gmail.com (S.-K.L.); believe1206920@gmail.com (J.-R.L.); sabrinablanco9779@gmail.com (S.D.B.); s970099307@gmail.com (H.-W.T.); applesell1999@gmail.com (J.-X.X.); ruby0109888@gmail.com (Y.-C.T.); tzean@mail.npust.edu.tw (Y.T.); 2 Department of Tropical Agriculture and International Cooperation, National Pingtung University of Science and Technology, Pingtung 912301, Taiwan

**Keywords:** agricultural management, biological control, PAMP-triggered immunity, stomata closure

## Abstract

When a plant is infected by a pathogen, endogenous immune responses are initiated. When the initiation of these defense responses is induced by a pathogen-associated molecular pattern (PAMP) of a pathogen, it is called PAMP-triggered immunity (PTI). Previous studies have shown that *Bacillus amyloliquefaciens* PMB05 can enhance PTI signals and improve disease control of bacterial soft rot and wilt in *Arabidopsis thaliana*. In the context of controlling bacterial wilt disease, the involvement of a mitogen-activated protein kinase (MAPK) signaling pathway has been established. Nevertheless, it remains unclear whether this pathway is also required for *B. amyloliquefaciens* PMB05 in controlling bacterial soft rot. In this study, *A. thaliana* ecotype Columbia (Col-0) and its mutants on a MAPK pathway-related pathway were used as a model and established that the ability of *B. amyloliquefaciens* PMB05 to control soft rot requires the participation of the MAPK pathway. Moreover, the enhancement of disease resistance by PMB05 is highly correlated with the activation of reactive oxygen species generation and stomata closure, rather than callose deposition. The spray inoculation method was used to illustrate that PMB05 can enhance stomatal closure, thereby restricting invasion by the soft rot bacterium. This control mechanism has also been demonstrated to require the activation of the MAPK pathway. This study demonstrates that *B. amyloliquefaciens* PMB05 can accelerate stomata closure via the activation of the MAPK pathway during PTI, thereby reducing pathogen invasion and achieving disease resistance against bacterial soft rot.

## 1. Introduction

With the increasing demand for food security in recent years, ensuring crop production while minimizing reliance on chemical fungicides for controlling plant diseases has become an important issue. Bacterial soft rot represents a significant threat to crops globally, affecting them during cultivation, transportation, and storage [1]. Pathogens in the Pectobacteriaceae family, such as *Pectobacterium* and *Dickeya* genera, are often the primary culprits in causing soft rot. These bacteria can produce large amounts of pectinase, leading to the breakdown of plant parenchyma tissue [2,3]. Consequently, these pathogens pose a severe threat to a wide range of vegetables and economically important ornamental plants [2,4]. In disease management, numerous studies have highlighted the effectiveness of beneficial microorganisms from the *Bacillus* genus, owing to their antagonistic activities [5,6,7], degradation of quorum sensing molecules [8,9], and plant immunity intensification [10].

In terms of enhancing plant immunity, previous studies have demonstrated that *Bacillus amyloliquefaciens* PMB05 effectively increases resistance against various diseases [11,12,13,14]. This acquired disease resistance is observed to be highly correlated with PAMP-triggered immunity (PTI), which serves as the plant’s primary defense mechanism [10,11,12]. Upon recognition of PAMPs by surface receptors, there is an influx of calcium ions that activates NADPH oxidase, resulting in the generation of reactive oxygen species (ROS) [15,16,17,18]. ROS function as signaling molecules, activating defense responses across the cell and triggering multiple defense pathways, such as calcium-dependent protein kinases (CDPKs) and mitogen-activated protein kinase (MAPK) pathways, which collectively confer disease resistance in plants [19,20,21].

The MAPK activated by PTI signals plays a crucial role in plant disease resistance, regulating various defense responses, such as ROS generation and stomatal closure [22,23,24]. In *Arabidopsis*, PTI triggered by flg22, a PAMP from a plant pathogenic bacteria, activates the MEKK1-MKK4/5-MPK3/6 pathway to defend against pathogen infection [19,25,26]. Our recent study demonstrates that the resistance of *Arabidopsis* to bacterial wilt improved by *B. amyloliquefaciens* PMB05 is associated with the activation of the MAPK-ROS pathway [11]. However, whether the PMB05-mediated control of bacterial soft rot also requires the MAPK pathway remains unclear. 

This study primarily investigates the role of the MAPK pathway in PMB05-mediated enhancement of bacterial soft rot resistance. Experiments were conducted using HrpN, a PAMP derived from soft rot bacteria, to assess the impact of PMB05 treatment on PTI signals such as ROS generation, callose deposition, and stomatal closure in *Arabidopsis*. Furthermore, *Arabidopsis* mutants deficient in the MAPK pathway were used to analyze the impact of PMB05’s effect on HrpN-induced PTI defense signals and resistance against bacterial soft rot. A spray inoculation model was also established to demonstrate PMB05’s ability to accelerate stomatal closure and reduce bacteria invasion in soft rot. MAPK mutants were used in this model to confirm that PMB05-induced disease reduction in soft rot incidence via stomatal closure requires the involvement of the MAPK pathway. Overall, the results provide compelling evidence that *B. amyloliquefaciens* PMB05 enhances resistance to bacteria soft rot primarily through the modulation of the MAPK pathway-regulated defense responses.

## 2. Materials and Methods

### 2.1. Growth Conditions of Plants and Bacteria

The plants utilized in this study were *Arabidopsis thaliana* ecotype Columbia (Col-0), and its *mekk1* (SALK_069473C), *mkk5* (SALK_047797C) and *mpk6* (SALK_062471C) mutants, which were obtained from the Arabidopsis Biological Resource Center (ABRC). Prior to the experiment, the non-sterilized seeds were sown in pots containing sterilized peat moss. After two weeks, germinated seedlings were individually transplanted into new pots. All plants were grown under controlled conditions at 25 °C with a light intensity of 100 μmol m^−2^ s^−1^ for 16 h/day in a plant growth chamber (Hipoint, Kaohsiung, Taiwan). The four-week-old plants were used in further analysis.

*Bacillus amyloliquefaciens* PMB05 and the bacterial soft rot pathogen, *Pectobacterium carotovorum* subsp. *carotovorum* Ecc17, were cultured on nutrient broth agar plates (NA, BD Difco™, Franklin Lakes, NJ, USA) at 28 °C for 48 h. Bacterial suspensions were then prepared and adjusted to an OD_620_ at 0.3 (around 3.0 × 10^8^ CFU/mL) with a sterilized phosphate buffer prior to conducting further experiments.

### 2.2. Preparation of HrpN Protein

The HrpN protein was prepared using the protocols described in our previous study [10]. Briefly, the *Escherichia coli* BL21 harboring pET-HrpN-Ecc17 was cultured in Luria-Bertani (LB) broth at 37 °C for 16 h. After the culture was transferred into fresh LB medium for an additional 4 h, 1 mM of isopropyl-β-D-thiogalactopyranoside (IPTG) was applied to induce protein expression, and the culture was left in incubation for an additional 16 h. In all procedures, ampicillin at 100 μg/mL was included in all LB broth formulations. The culture was centrifuged at 8000× *g* for 5 min at 4 °C, and the pellet was resuspended with 0.1 M phosphate buffer (pH 8.0). The suspension was then sonicated and treated at 100 °C for 10 min. Finally, the supernatant obtained by centrifugation at 10,000× *g* for 10 min treatment was used as the HrpN protein extract for subsequent experiments. 

### 2.3. Disease Control Assay

The disease control assay for bacterial soft rot in *Arabidopsis* plants treated with *B. amyloliquefaciens* PMB05 was conducted using the spray inoculation method. First, the bacterial suspensions of *B. amyloliquefaciens* PMB05 and *P. carotovorum* subsp. *carotovorum* Ecc17 were prepared. These bacterial suspensions were mixed in equal proportions (1:1 *v*/*v*) and sprayed onto the leaves of the plants. The inoculated plants were then placed in a humidified container and placed in a growth chamber at 22 °C. According to the symptoms observed after inoculation, the scale used to analyze the disease severity was divided into four levels (0, no symptom; 1, soft rot leaf area below 25%; 2, 25–50% of soft rot leaf area; 3, 50–75% of soft rot leaf area; 4, more than 75% of soft rot leaf area). The disease severity was calculated using the following formula: Disease severity = [(0 × N_0_ + 1 × N_1_ + 2 × N_2_ + 3 × N_3_ + 4 × N_4_)/(4 × N_total_)] × 100%, where N_0_–N_4_ represents the number of plants in each different disease scales and N_total_ represents total number of plants inoculated in the treatment. The results were collected from 30 inoculated leaves as repeats in each treatment.

### 2.4. ROS Generation and Callose Deposition Assay

To assess whether *B. amyloliquefaciens* PMB05 enhances HrpN-activated ROS generation and callose deposition in MAPK mutants, the assays were performed following standard procedures [12]. Before infiltration, mixtures containing equal volumes of HrpN, PMB05 bacterial suspension, and phosphate buffer were prepared for distinct treatments. In the treatments containing HrpN, its final protein concentration was 0.5 mg/mL. ROS generation was assayed using 20 μM of 2′,7′-dichlorodihydrofluorescein diacetate (Molecular Probes, Eugene, OR, USA) to stain infiltrated leaves. The stained leaves were examined under a fluorescence microscope with a GFP filter cube (Leica Microsystems, Wetzlar, Germany). Callose deposition was examined by staining the infiltrated leaves with 0.01% aniline blue (Sigma, St. Louis, MO, USA), which were then examined under a fluorescence microscope with a DAPI filter cube (Leica Microsystems, Wetzlar, Germany). The intensity of ROS generation and callose deposition was determined using ImageJ software ver. 1.54j ( https://imagej.net/ij/, accessed on 1 July 2024) under consistent threshold conditions. Each treatment included ten replicates.

### 2.5. Stomatal Closure Assay

To assess the effect of *B. amyloliquefaciens* PMB05 on stomatal closure during PTI induction in *Arabidopsis* mutants, moisture-treated plants were analyzed. Four-week-old *Arabidopsis* mutants were placed in a sealed container with paper towels soaked in sterile water to maintain humidity for 24 h before the assay. Three hours after the lights were turned on, each treatment solution was sprayed onto the leaf surface, with final concentrations of HrpN protein being 0.5 mg/mL and the OD_620_ of the PMB05 bacterial suspension being approximately 0.15. Leaf samples were collected at 15, 30, 60, and 90 min post-treatment, and images of stomata were captured using a microscope (Leica Microsystems, Wetzlar, Germany). Stomatal aperture analysis was performed using ImageJ software. For each treatment, 100 stomatal apertures were measured as replicates.

### 2.6. Population Assay of Soft Rot Bacteria Invading Leaf Tissues

To investigate whether *B. amyloliquefaciens* PMB05 reduces the invasion of soft rot bacteria into plant tissues and its potential association with the MAPK pathway, wild-type Col-0 and MAPK pathway-related mutants were used. The spray inoculation method described previously was used. Inoculated plants were placed in a humidified container and kept in a growth chamber at 22 °C. Samples were collected post-inoculation to assess the population of soft rot bacteria. To determine the total soft rot bacteria in the leaves, leaf tissues were ground with sterile water at a ratio of 100 µL/cm^2^, and the resulting suspension was serially diluted and plated on nutrient agar (NA) plates for bacterial counting. To analyze the population of soft rot bacteria within leaf tissues, inoculated leaves were surface sterilized by soaking in 1% sodium hypochlorite solution for 2 min, followed by three washes with sterile water for 1 min before sampling. Each treatment was conducted with 5 leaves as replicates.

### 2.7. Data Analysis

Statistical analyses were performed using SPSS Statistics software for Windows, version 25 (IBM Corp., Armonk, NY, USA). Analysis of variance (ANOVA) was used to assess differences between treatments. Post hoc tests (Tukey’s HSD) or *t*-tests were performed to analyze significant differences between treatments (*p* < 0.05). 

## 3. Results

### 3.1. Effect of Bacillus amyloliquefaciens PMB05 on the Control of Soft Rot in the MAPK Mutants of Arabidopsis thaliana

**Figure 1:**
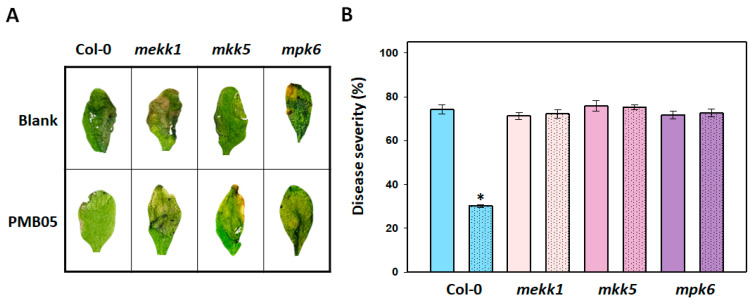
Effect of *Bacillus amyloliquefaciens* PMB05 on the control of bacterial soft rot in *Arabidopsis thaliana* mutants. Panel (**A**) shows the symptom appearance of bacterial soft rot, and Panel (**B**) shows the disease severity of bacterial soft rot at 2 days post-inoculation. The inoculation of bacterial soft rot was performed by the spray method on 4-week-old *mekk1*, *mkk5*, and *mpk6* mutants as previously described in the Materials and Methods. After inoculation, the indexes of soft rot symptoms were evaluated at 2 days post-inoculation. Unmarked columns represent plants inoculated with *Pectobacterium carotovorum* subsp. *carotovorum* Ecc17 alone, while columns with dotted patterns represent plants inoculated with the co-treatment of *B. amyloliquefaciens* PMB05 and *P. carotovorum* subsp. *carotovorum* Ecc17. The * above the columns indicate a significant difference compared with blank treatment based on a *t*-test (*p* < 0.05).

To understand whether the MAPK pathway is required for *B. amyloliquefaciens* PMB05 to enhance resistance against soft rot disease in *Arabidopsis*, inoculation assays were conducted on *mekk1*, *mkk5*, and *mpk6* mutants. After inoculation with *P. carotovorum* subsp. *carotovorum* Ecc17, rapid development of soft rot symptoms was observed in Col-0, *mekk1*, *mkk5*, and *mpk6* plants at 2 days post-inoculation. Treatment with the bacterial suspension of *B. amyloliquefaciens* PMB05 reduced the occurrence of soft rot only in Col-0 plants (Figure 1A), resulting in a decrease in disease severity from 74.2% to 30.1% at 2 days post-inoculation (Figure 1B). This enhancement of disease resistance by *B. amyloliquefaciens* PMB05 was absent in the *mekk1*, *mkk5*, and *mpk6* mutants, where disease severities were 72.2%, 75.1%, and 72.6%, respectively.

### 3.2. Intensification of Plant Immune Signals by Bacillus amyloliquefaciens PMB05 in MAPK-Deficient Mutants

**Figure 2:**
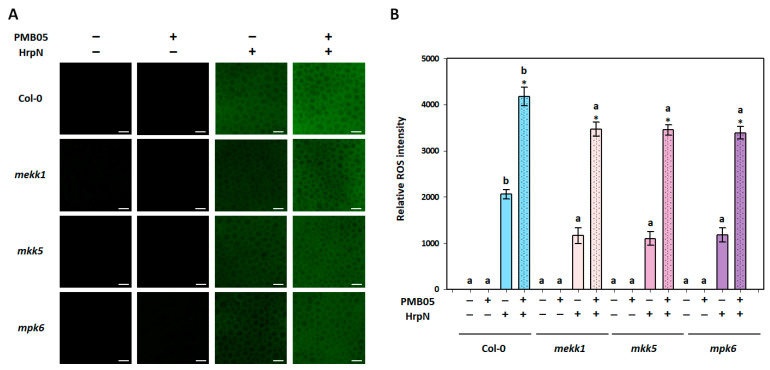
Effect of *Bacillus amyloliquefaciens* PMB05 on HrpN-induced reactive oxygen species (ROS) generation in *Arabidopsis thaliana* mutants. Panel (**A**) shows the fluorescent images, with white bars indicating 50 μm in length. Panel (**B**) shows the quantification of relative fluorescent intensities of HrpN-induced ROS generation in *mekk1*, *mkk5*, and *mpk6* mutants. The symbols “+” and “−” indicate the inclusion or exclusion of treatments with HrpN and PMB05, respectively. Different letters on the columns indicate significant differences among the four types of plants (Col-0, *mekk1*, *mkk5*, and *mpk6*) for the same treatment (Tukey’s HSD test, *p* < 0.05). Asterisks (*) above the co-treatment columns indicate a significant difference compared to the HrpN-only treatment within the same plant type (*t*-test, *p* < 0.05).

To investigate whether the *B. amyloliquefaciens* PMB05-mediated enhancement of plant immune responses is influenced by the MAPK pathway, assays were conducted on ROS generation and callose deposition in *mekk1*, *mkk5*, and *mpk6* mutants. In the ROS generation assay, the fluorescent signal of ROS was induced by HrpN in all *Arabidopsis* plants, while PMB05 alone did not. However, the fluorescent intensities of HrpN-induced ROS were significantly lower in all mutants compared to Col-0 plants. Although HrpN-induced ROS signals were greatly enhanced by *B. amyloliquefaciens* PMB05 in all plants, the ROS signals generated in *mekk1*, *mkk5*, and *mpk6* mutants were significantly lower than those in Col-0 plants (Figure 2).

**Figure 3:**
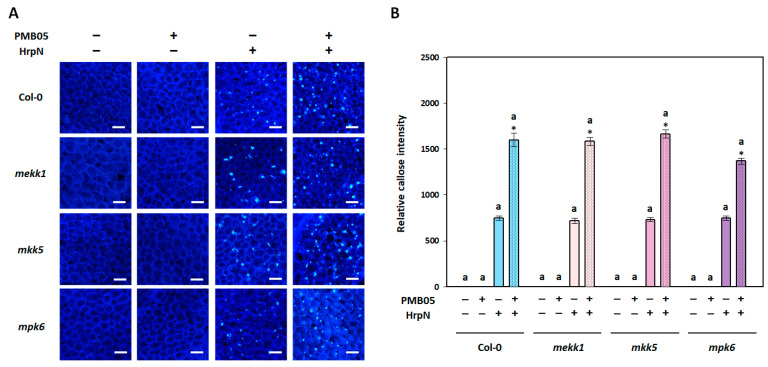
Effect of *Bacillus amyloliquefaciens* PMB05 on HrpN-induced callose deposition in *Arabidopsis thaliana* mutants. Panel (**A**) shows fluorescent images, with white bars indicating 50 μm in length. Panel (**B**) shows the quantification of the relative fluorescent intensities of HrpN-induced callose deposition in *mekk1*, *mkk5*, and *mpk6* mutants. The symbols “+” and “−” indicate the inclusion and exclusion of treatments with HrpN and PMB05, respectively. Different letters on the columns indicate significant differences among the four plant types (Col-0, *mekk1*, *mkk5*, and *mpk6*) for the same treatment (Tukey’s HSD test, *p* < 0.05). Asterisks (*) above the co-treatment columns indicate a significant difference compared to the HrpN-only treatment within the same plant type (*t*-test, *p* < 0.05).

In the callose deposition assay, the results showed that only HrpN, not PMB05, induced the production of callose fluorescent signals in all plants. However, the callose fluorescence signals induced by HrpN in mutants were not significantly different from those in Col-0 plants. In addition, *B. amyloliquefaciens* PMB05 significantly intensified the HrpN-induced callose fluorescence signals in Col-0 plants. This intensification was also observed in *mekk1*, *mkk5*, and *mpk6* mutants with no significant difference from Col-0 plants (Figure 3).

### 3.3. HrpN-Induced Stomatal Closure Affected by B. amyloliquefaciens PMB05

**Figure 4:**
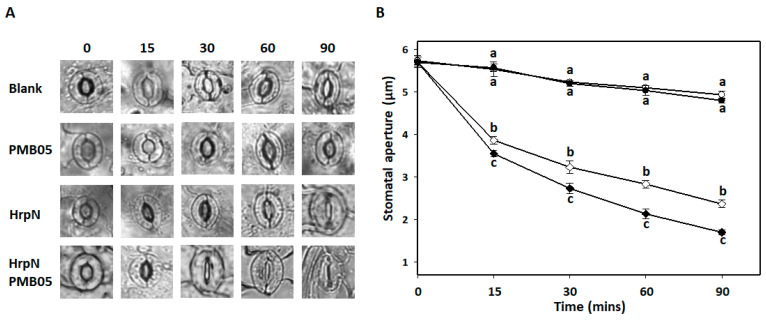
Effect of *Bacillus amyloliquefaciens* PMB05 on HrpN-induced stomatal closure in *Arabidopsis thaliana*. Panel (**A**) shows microscopic images of stomatal guard cells during exposure to different PMB05 and HrpN treatments. Panel (**B**) shows the changes in stomatal aperture length at different time points in the *A. thaliana* Col-0 plant. Lines with symbols of open circles, closed circles, open diamonds, and closed diamonds indicate the treatment application with Tris (blank), bacterial suspension of *B. amyloliquefaciens* PMB05 alone, HrpN alone, and PMB05/HrpN co-treatment, respectively. Different letters on the symbols indicate significant differences between treatments at each time point based on Tukey’s HSD test (*p* < 0.05).

To investigate the capability of *B. amyloliquefaciens* PMB05 to accelerate stomatal closure during PTI activation, an analysis of HrpN-induced stomatal closure was conducted. The results demonstrated a gradual reduction in the stomatal aperture within 90 min after treatment with a Tris buffer (blank) or PMB05 alone, with no significant difference observed between these two treatments. Upon induction with HrpN, a rapid reduction in the stomatal aperture and a tighter closure over time were observed. Quantitative analysis revealed that stomatal apertures at all time points starting from 15 min post-HrpN treatment were significantly smaller compared to blank or PMB05-alone treatment. Further analysis of PMB05 on HrpN-induced stomatal closure demonstrated that PMB05 treatment accelerated and tightened stomatal closure, significantly differing from HrpN treatment alone. The stomatal apertures measured after 90 min after treatment with blank, PMB05, HrpN alone, and HrpN/PMB05 mixture were 5.1 μm, 5.0 μm, 2.8 μm, and 1.7 μm, respectively (Figure 4).

### 3.4. Restriction of Bacillus amyloliquefaciens PMB05 on the Invasion of Soft Rot Bacteria into Arabidopsis Leaves

**Figure 5:**
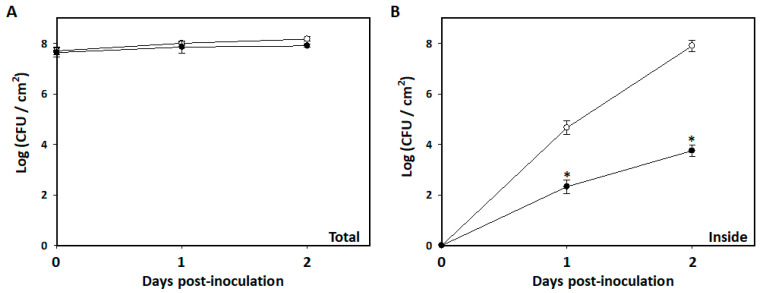
Effect of *Bacillus amyloliquefaciens* PMB05 on population dynamics in *Arabidopsis thaliana*. Panel (**A**) shows the total population and panel (**B**) shows inside tissue populations of *B. amyloliquefaciens* PMB05 in the *A. thaliana* Col-0 plant. The symbols of open circles and closed circles indicate the treatment with *Pectobacterium carotovorum* subsp. *carotovorum* Ecc17 alone and its co-treatment with *B. amyloliquefaciens* PMB05, respectively. The * on the symbols indicates significant differences between the two treatments at each time point based on a *t*-test (*p* < 0.05).

To determine whether the enhanced disease resistance by *B. amyloliquefaciens* PMB05 is associated with restricting the invasion of *P. carotovorum* subsp. *carotovorum*, changes in bacterial population within inoculated leaves were evaluated. After the spray inoculation with *P. carotovorum* subsp. *carotovorum* Ecc17, the total bacterial population on leaves showed minimal change within two days. In both blank and PMB05 treatments, the total population of Ecc17 on the leaf was approximately 1.0 × 10^8^ CFU/cm^2^ (Figure 5A). Further analysis of the bacterial population inside the leaf tissues revealed no detectable Ecc17 in both treatments 30 min after spraying. However, the bacterial population of Ecc17 in both treatments gradually increased at one and two days post-inoculation, with significantly lower numbers of Ecc17 invading leaves in the PMB05 treatment compared to the blank treatment. Specifically, the bacterial populations of Ecc17 inside leaves were 8.0 × 10^7^ and 5.6 × 10^3^ CFU/cm^2^ in the blank and PMB05 treatments, respectively, two days post-inoculation (Figure 5B). 

### 3.5. Restriction of Bacillus amyloliquefaciens PMB05 on Soft Rot Bacteria Invasion in Arabidopsis thaliana MAPK Mutants

**Figure 6:**
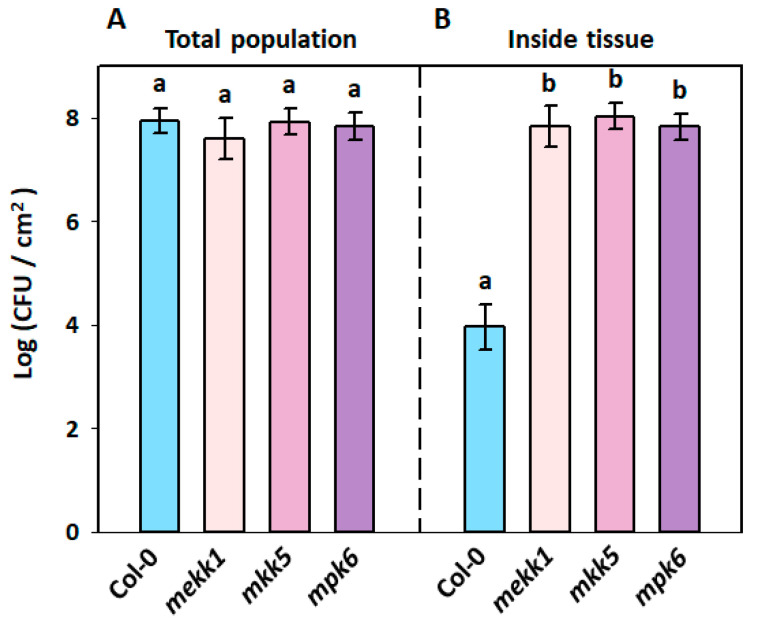
Effect of *Bacillus amyloliquefaciens* PMB05 on population dynamics in *Arabidopsis thaliana* mutants. Panel (**A**) shows the total population, and panel (**B**) shows the inside tissue populations in *A. thaliana* plants. All of the treatments were carried out with the co-treatment of *Pectobacterium carotovorum* subsp. *carotovorum* Ecc17 and *B. amyloliquefaciens* PMB05. Different letters on the bars indicate significant differences among four plant types based on Tukey’s HSD test (*p* < 0.05).

To further investigate whether the reduction in soft rot bacterial invasion by PMB05 is related to the MAPK pathway, a spray inoculation method was used to analyze bacterial population changes of soft rot bacteria in the leaves of *mekk1*, *mkk5*, and *mpk6* mutants. The results showed total bacterial populations of Ecc17 on the leaves of Col-0, *mekk1*, *mkk5*, and *mpk6* at 2 days post-inoculation, with 7.9 × 10^7^, 3.6 × 10^7^, 7.2 × 10^7^, and 5.6 × 10^7^ CFU/cm^2^, respectively, with no significant differences among plants (Figure 6A). However, analysis of bacterial load inside leaves following surface disinfection showed that the bacterial populations of Ecc17 in the leaves of Col-0, *mekk1*, *mkk5*, and *mpk6* at 2 days post-inoculation were 7.9 × 10^3^, 5.2 × 10^7^, 1.3 × 10^8^, and 6.3 × 10^7^ CFU/cm^2^, respectively. The bacterial populations of Ecc17 inside the leaves of all MAPK mutants were significantly higher than those inside the leaves of Col-0 plants (Figure 6B).

### 3.6. Bacillus amyloliquefaciens PMB05-Accelerated Stomatal Closure upon HrpN Induction in MAPK Mutants

**Figure 7:**
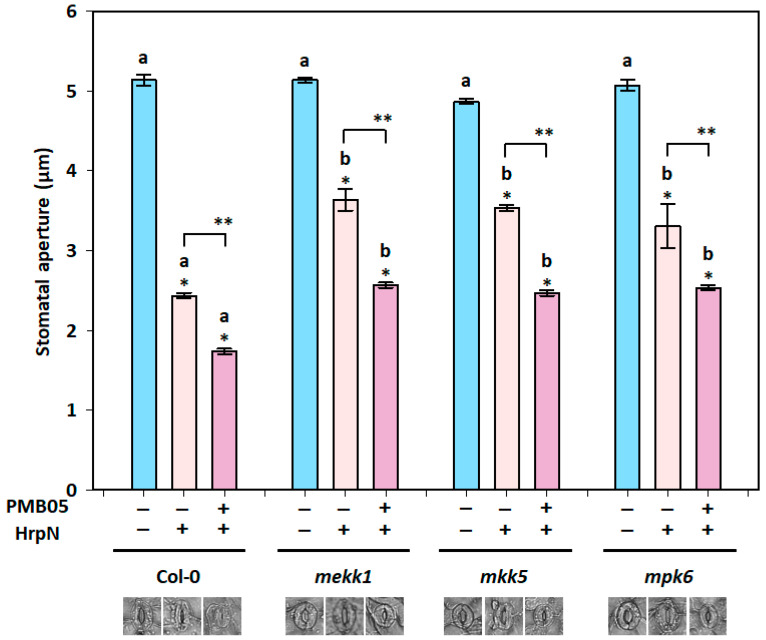
Effect of *Bacillus amyloliquefaciens* PMB05 on HrpN-induced stomatal closure in *Arabidopsis thaliana* mutants. The stomatal apertures in *mekk1*, *mkk5*, and *mpk6* mutants were evaluated at 90 min post-treatment. The symbols “+” and “−” indicate the inclusion and exclusion of HrpN and *B. amyloliquefaciens* PMB05 treatments. Different letters on the columns indicate significant differences among the four plant types (Col-0, *mekk1*, *mkk5*, and *mpk6I*) for the same treatment (Tukey’s HSD test, *p* < 0.05). Single asterisks (*) indicate significant differences compared to the blank treatment within the same plant type (*t*-test, *p* < 0.05), and double asterisks (**) indicate significant differences between treatments (*t*-test, *p* < 0.05).

To clarify the correlation between the reduction in soft rot bacterial invasion on leaves by *B. amyloliquefaciens* PMB05 and the requirement of MAPK pathway function during PTI intensification, *mekk1*, *mkk5*, and *mpk6* mutants were used to assess stomatal closure upon HrpN induction. The results revealed that 90 min after treatment with HrpN alone, stomatal apertures in *mekk1*, *mkk5,* and *mpk6* mutants were 3.6 μm, 3.5 μm, and 3.3 μm, respectively, which were significantly larger than the 2.4 μm observed in Col-0 plants. Meanwhile, co-treatment with HrpN and *B. amyloliquefaciens* PMB05 resulted in smaller stomatal apertures on all plants compared to HrpN treatment alone. However, stomatal apertures in *mekk1*, *mkk5,* and *mpk6* mutants were 2.6 μm, 2.5 μm, and 2.5 μm, respectively, and all were significantly larger than the 1.7 μm observed in Col-0 plants (Figure 7). 

## 4. Discussion

In the battle against pathogenic microorganisms, plants were selected for having mechanisms to detect and respond to pathogen invasion. In this process, PTI serves as the plant’s first line of defense against disease [27,28,29]. Once a plant recognizes pathogenic bacteria, further defense responses are commonly signaled through the MEKK1-MKK4/5-MPK3/6 pathway [19,30]. Previous studies have shown that *B. amyloliquefaciens* PMB05 can intensify PTI signals induced by PAMPs, like flg22 and harpin, thereby effectively increasing resistance to various plant diseases [10,11,12,13,14]. Studies using bacterial wilt as a model have further demonstrated the necessity of a coordinated MAPK and ROS regulatory system for the disease control efficacy of *B. amyloliquefaciens* PMB05 [11]. However, whether this mechanism extends to the control of phyllosphere diseases remains unclear. Therefore, this study investigates whether the involvement of the MAPK pathway is necessary for *B. amyloliquefaciens* PMB05 to enhance PTI and confer disease resistance against bacterial soft rot.

Initially, the efficacy of *B. amyloliquefaciens* PMB05 in controlling bacterial soft rot in *mekk1*, *mkk5*, and *mpk6* mutants was analyzed, and the results showed a complete loss of control effect in these mutants. These findings suggest that *B. amyloliquefaciens* PMB05 indeed requires signal transduction from MEKK1 to MKK5 to MPK6 to enhance *Arabidopsis* resistance against bacterial soft rot. Further analysis of ROS generation in response to HrpN from soft rot bacteria showed that the ROS signals were significantly suppressed in all MAPK mutants. However, the fluorescence signal for callose deposition was still induced and further intensified by *B. amyloliquefaciens* PMB05 in these mutants upon HrpN induction. These results imply that ROS generation is closely linked to the MAPK pathway rather than the callose deposition among the various defense signals elicited during PTI. These outcomes are consistent with studies involving PopW from the bacterial wilt pathogen in MAPK mutants [11]. While *B. amyloliquefaciens* PMB05 can enhance callose deposition induced by PAMPs and activate PTI, the findings presented here suggest that the callose deposition may not contribute significantly to the control of bacterial diseases, as observed in both bacterial wilt and soft rot cases. These findings are consistent with studies where callose deposition induced by flg22 treatment in *mekk1* and *mpk6* mutants showed diminished effectiveness [31,32]. This also supports the notion proposed by Luna et al. that callose induction can be regulated by multiple signaling pathways [33]. Therefore, it can be inferred that the control of soft rot by *B. amyloliquefaciens* PMB05 indeed requires the MAPK signaling pathway.

In the complex defense network regulated by the MAPK pathway, stomatal closure regulated by it serves as a pivotal indicator of disease resistance [34,35,36]. Stomata, facilitating gas exchange and water evaporation, also serve as natural entry points for bacterial invasion [37,38,39]. Thus, investigating whether *B. amyloliquefaciens* PMB05 accelerates stomatal closure through MAPK signaling to reduce soft rot bacteria invasion warranted further exploration. In this study, the results confirmed that HrpN from soft rot bacteria induced stomatal closure, and subsequent treatment with *B. amyloliquefaciens* PMB05 accelerated and tightened this closure. Inoculation analysis revealed that PMB05 treatment significantly reduced disease severity, with soft rot symptoms predominantly initiating at leaf margins. Analysis of the population dynamics of soft rot bacteria also indicated that PMB05 treatment reduced bacterial invasion into leaves. In the absence of a decrease in the total bacterial load on the leaves, the present findings suggest that the control of bacterial soft rot by *B. amyloliquefaciens* PMB05 is linked to accelerated stomatal closure. Since bacterial soft rot invades plant tissues through natural openings [40], the use of highly concentrated bacterial suspensions for spray inoculation in this study effectively simulated the natural entry of soft rot bacteria through stomata and explored the role of *B. amyloliquefaciens* PMB05 in controlling soft rot. To further explain that the regulation of stomatal closure by PMB05 is indeed associated with the MAPK pathway, the bacterial population changes of soft rot bacteria post-inoculation in MAPK mutants were analyzed. The results demonstrated significantly higher bacterial populations in the leaves of all MAPK mutants compared to wild-type plants. Moreover, the enhanced HrpN-induced stomatal closure by *B. amyloliquefaciens* PMB05 was significantly reduced in MAPK mutants, implying that enhancing stomatal closure by PMB05 to limit pathogen invasion requires the MAPK pathway. The results from MAPK mutants not only confirm that *B. amyloliquefaciens* PMB05 enhances stomatal closure during PTI induction, requiring the participation of the MAPK pathway, but also suggest the involvement of other pathways in stomatal closure during this process. For instance, the plant hormone abscisic acid (ABA) also plays an important role in plant immunity [38,41,42]. In the ABA synthesis-deficient mutant *aba*3-1, reduced ABA function diminishes the effect of PAMP and *Pseudomonas syringae* pv. *tomato* DC3000-induced stomatal closure [38]. Furthermore, MAPK inhibitors can inhibit ABA-induced stomatal closure [43], suggesting a relationship between MAPK and ABA signaling in guard cells. Moreover, Su et al. found that both the MPK3/MPK6 cascade and ABA are crucial signaling pathways in stomatal closure [44]. According to these results, it is plausible that the MAPK and ABA pathways may independently or cooperatively regulate stomatal closure. Reports indicate that the MAPK pathway may regulate multiple defense signaling pathways through different transcription factors [45]. Whether *B. amyloliquefaciens* PMB05 can enhance stomatal closure through other regulatory pathways during the process of intensifying plant immunity still requires further investigations.

This study demonstrates that *B. amyloliquefaciens* PMB05 enhances disease resistance in *A. thaliana* against bacterial soft rot through the activation of the MAPK signaling pathway. The MAPK signaling pathway is crucial for accelerating stomatal closure and restricting pathogen invasion, playing a pivotal role in plant defense mechanisms. Our findings show that PMB05-mediated enhancement of resistance is primarily associated with ROS generation, while callose deposition may not significantly contribute to disease resistance. Together, these results indicate that *B. amyloliquefaciens* PMB05 enhances plant immunity by modulating the MAPK signaling pathway, thereby enhancing both physical and genetic defense responses to improve overall plant disease resistance.

Further exploration of additional regulatory pathways involved in PMB05-mediated disease resistance would be an important next step. For instance, investigating the role of the ABA pathway and its potential crosstalk with MAPK signaling, particularly in stomatal closure and overall plant immunity, could offer new insights. Such studies would broaden the understanding of the molecular mechanisms underlying PMB05’s biocontrol efficacy, potentially leading to more efficient strategies for controlling bacterial soft rot and other plant diseases. 

## Data Availability

The original contributions presented in the study are included in the article, further inquiries can be directed to the corresponding author.
